# Functionalization of SF/HAP Scaffold with GO-PEI-miRNA inhibitor Complexes to Enhance Bone Regeneration through Activating Transcription Factor 4: Erratum

**DOI:** 10.7150/thno.86033

**Published:** 2023-05-30

**Authors:** Lingling Ou, Yong Lan, Zhiqiang Feng, Longbao Feng, Junjie Yang, Yu Liu, Liming Bian, Jiali Tan, Renfa Lai, Rui Guo

**Affiliations:** 1 The First Affiliated Hospital of Jinan University, Guangzhou, 510632, China; 2 Beogene Biotech (Guangzhou) CO., LTD, Guangzhou 510663, China; 3 Guangzhou Chuangseed Biomedical Materials CO., LTD, Guangzhou 510663, China; 4 Department of Biomedical Engineering, The Chinese University of Hong Kong, Hong Kong SAR, China; 5 Department of Orthodontics, Guanghua School of Stomatology, Hospital of Stomatology, Sun Yat-sen University and Guangdong Provincial Key Laboratory of Stomatology, Guangzhou, 510055, China; 6 Key Laboratory of Biomaterials of Guangdong Higher Education Institutes, Guangdong Provincial Engineering and Technological Research Center for Drug Carrier Development, Department of Biomedical Engineering, Jinan University, Guangzhou 510632, China

The authors regret that the original version of our paper, unfortunately, contained incorrect pictures in Figure 3E and Figure 7B. The cell nucleus image of miR-inhibitor-Cy3 group was mistakenly used for the control group in Figure 3E when uploading the images. The first and the sixth western blotting bands were the same band in Figure 7B, this error had occurred while compiling the affected figure parts.

The correct version of Figure 3E and Figure 7B are shown below.

The correction made in this erratum does not affect the original data and conclusions. The authors apologize for any inconvenience that the errors may have caused.

## Figures and Tables

**Figure 3:**
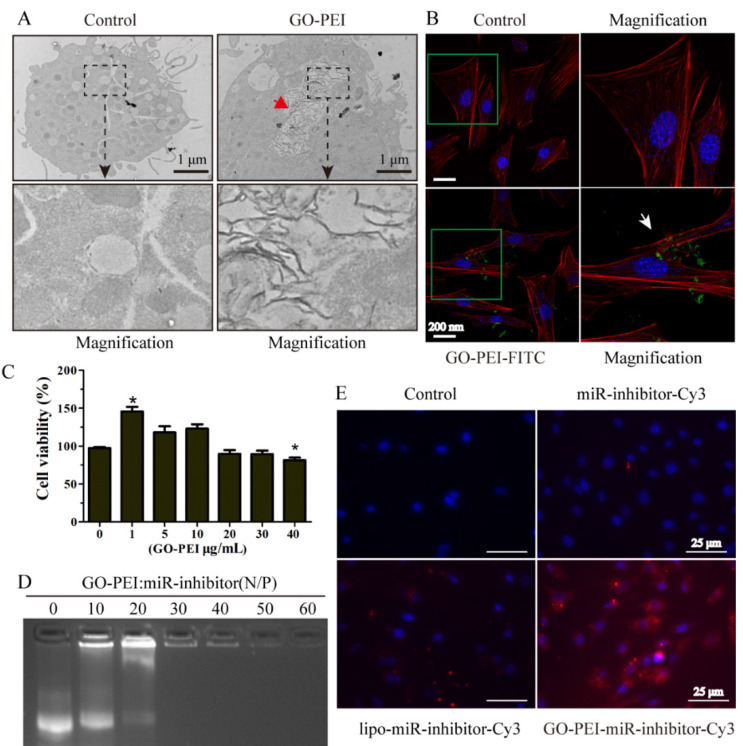
** Evaluation of GO-PEI complex biocompatibility and delivery effectiveness.** (A) TEM images of non-GO-PEI cells (named as control) and GO-PEI-induced cells (the concentration of GO-PEI in the culture medium was 3 μg/mL). The images below each group were the enlarged images of the black squares. The red arrowhead indicates GO-PEI inside the cells. Scale bars: 1 μm. (B) Fluorescent images of FITC-labeled GO-PEI (green) within MC3T3-E1 cells are shown. The cell cytoskeleton was stained with phalloidin (red), and the nuclei were stained with DAPI (blue). The FITC-labeled GO-PEI inside the cells was indicated by the white arrowhead. Scale bars: 200 nm. The images on the right of each group are the enlarged images of green squares. (C) The viability of MC3T3-E1 cells was measured by the CCK-8 assay after incubation with various concentrations of GO-PEI for 48 h. *p < 0.05. (D) A gel retardation assay of the mixture of GO-PEI and miRNA at different N/P ratios (0, 10, 20, 30, 40, 50 and 60). (E) Fluorescent images for GO-PEI complex delivery of Cy3-labeled miR-214 inhibitor after 24 h of incubation. miR-214 was labeled using Cy3 (orange red), and the cell nuclei were stained with DAPI (blue).

**Figure 7:**
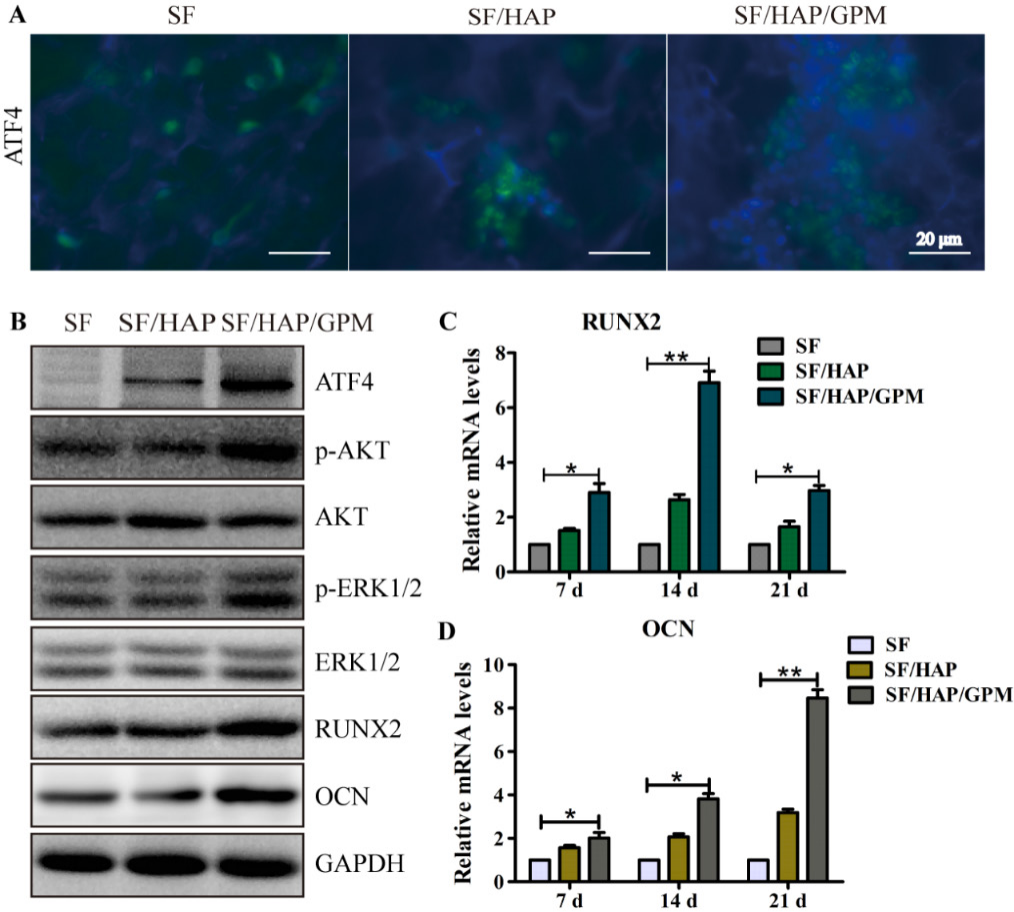
** The signaling pathway by which SF/HAP/GPM scaffolds promote osteogenesis.** (A) Immunofluorescence staining of ATF4 in MC3T3-E1 cells on SF, SF/HAP and SF/HAP/GPM scaffolds. Scale bars: 20 μm. (B) The expression levels of ATF4, p-Akt, Akt, p-ERK1/2, ERK1/2, RUNX2 and OCN in MC3T3-E1 cells incubated on SF, SF/HAP and SF/HAP/GPM scaffolds for 14 days. (C and D) The expression levels of RUNX2 and OCN on SF, SF/HAP and SF/HAP/GPM scaffolds were evaluated by qRT-PCR.

